# Mental Health Inequities Amid the COVID-19 Pandemic: Findings From Three Rounds of a Cross-Sectional Monitoring Survey of Canadian Adults

**DOI:** 10.3389/ijph.2022.1604685

**Published:** 2022-07-21

**Authors:** Emily K. Jenkins, Allie Slemon, Chris Richardson, Javiera Pumarino, Corey McAuliffe, Kimberly C. Thomson, Trevor Goodyear, Zachary Daly, Liza McGuinness, Anne Gadermann

**Affiliations:** ^1^ School of Nursing, University of British Columbia, Vancouver, BC, Canada; ^2^ School of Population and Public Health, University of British Columbia, Vancouver, BC, Canada

**Keywords:** mental health, public health, COVID-19, survey, structural vulnerability, inequities, syndemics theory

## Abstract

**Objectives:** Adverse mental health impacts of the COVID-19 pandemic are well documented; however, there remains limited data detailing trends in mental health at different points in time and across population sub-groups most impacted. This paper draws on data from three rounds of a nationally representative cross-sectional monitoring survey to characterize the mental health impacts of COVID-19 on adults living in Canada (*N* = 9,061).

**Methods:** Descriptive statistics were used to examine the mental health impacts of the pandemic using a range of self-reported measures. Multivariate logistic regression models were then used to quantify the independent risks of experiencing adverse mental health outcomes for priority population sub-groups, adjusting for age, gender, and survey round.

**Results:** Data illustrate significant disparities in the mental health consequences of the pandemic, with inequitable impacts for sub-groups who experience structural vulnerability related to pre-existing mental health conditions, disability, LGBTQ2+ identity, and Indigenous identity.

**Conclusion:** There is immediate need for population-based approaches to support mental health in Canada and globally. Approaches should attend to the root causes of mental health inequities through promotion and prevention, in addition to treatment.

## Introduction

The novel coronavirus (COVID-19) pandemic has contributed profound negative impacts to the mental health of populations globally [1–3], with elevated rates of depression, anxiety, post-traumatic stress symptoms, and suicidal thoughts and behaviours [4–7]. For example, in a recent systematic review and meta-analysis published in the Lancet and drawing on data from 204 countries, the prevalence of depressive and anxiety disorders were determined to have increased by approximately 28% and 26%, respectively [7]. Beyond clinical conditions, elevated levels of fear, stress, and worry are also undermining population mental health and wellbeing [8]. Moreover, data have documented that those who experience structural vulnerability face inequitable risk for adverse mental health outcomes, likely a result of their relative positions within intersecting power hierarchies that constrain access to determinants of good health [1]. For example, mental health impacts attributed to the pandemic have been particularly pronounced among people who are racialized [9]; Indigenous [10]; lesbian, gay, bisexual, transgender, Two-Spirit, or queer (LGBT2Q+) [11, 12]; experiencing poverty [3]; and living with pre-existing mental health conditions [13] or disability [14].

The mental health impacts observed during COVID-19 are aligned with those identified during previous virus outbreaks such as SARS and are expected in the context of pandemic conditions [15]. However, a notable difference exists – previous outbreaks resolved relatively quickly, whereas the global spread and duration of COVID-19 is unprecedented in recent times. Concerningly, evidence suggests that as the pandemic endures, population mental health may further deteriorate [16, 17] and exacerbate mental health inequities for certain sub-groups. Researchers have further cautioned that these impacts are likely to persist beyond the resolution of this pandemic and may lead to chronic mental health conditions for some individuals [18].

Despite the widespread and inequitable mental health consequences of the pandemic, there is limited data characterizing population mental health at different points in time. The majority of research conducted to date has comprised single studies, providing snapshots of population mental health impacts among the general population or population sub-groups [4]. There remains a priority need for evidence examining population mental health trends to guide policy, public health responses, resource allocation, and health service delivery [19, 20]. Crucially, such explorations must also illuminate the characteristics associated with elevated risk, as this disaggregation provides a more comprehensive picture [9, 21]. The current investigation responds to this evidence need. Specifically, it presents an analysis of data from three rounds of a repeated cross-sectional survey designed to monitor the mental health impacts of COVID-19 for adults living in Canada, with a particular focus on deteriorations in mental health, suicidal ideation and use of substances to cope. The selection of these outcomes was guided by emerging literature identifying these constructs as priority areas of interest (e.g., 2,5,9). The research objectives are twofold: 1) to characterize the mental health impacts of the pandemic according to self-reported measures of mental health and substance use; and 2) to quantify the independent risks of experiencing adverse mental health outcomes for structurally vulnerable population sub-groups, while adjusting for age, gender and survey round. Our aim is to contribute to a better understanding of population mental health trends over the course of the pandemic and provide evidence to inform targeted responses to maximize benefit.

### Theoretical Positioning

This study draws on syndemics theory and prior evidence regarding structural vulnerability and health inequities. First, we follow Bourgois et al. [22] and define “structural vulnerability” as the inequitable burden of risk for negative health outcomes stemming from individuals’ locations within intersecting socio-economic and political power hierarchies. Second, we utilize syndemics theory as a framework for understanding how mental health disparities among structurally vulnerable groups are created and perpetuated by the COVID-19 pandemic [20, 23, 24]. Proposed by Singer in the 1990s to support understandings about the transmission of HIV within structurally vulnerable communities, the theory draws attention to the “synergistic interaction” between disease, other health conditions, and the social, political, economic, and environmental factors influencing health and well-being [25]. Indeed, the potential for syndemic effects of COVID-19 are demonstrated by the aggregation of the virus alongside non-communicable diseases—including mental health conditions—within the context of inequitable societal contexts that increase risks for virus transmission, exacerbate underlying illness, and worsen health and social inequities [20, 23, 24]. Already, the COVID-19 pandemic has been identified as syndemic in some contexts due to disparities in COVID-19 prevalence and physical health consequences among structurally vulnerable populations [23, 24]. This paper extends this domain of inquiry by analyzing the mental health impacts of the COVID-19 pandemic, focusing particular attention to how syndemic structural vulnerabilities intersect to shape mental health inequities.

## Methods

### Survey Background

This study is supported by a partnership between researchers from the University of British Columbia (UBC) and the Canadian Mental Health Association (CMHA), as well as international collaboration with the Mental Health Foundation of the United Kingdom. Our research team brings a diversity of life experiences and social identities to this work, including members who identify as living with disability and/or mental health challenges, who are racialized and/or recent immigrants to Canada, and who identify as LGBTQ2+.

### Sample and Procedures

Data are drawn from the first three rounds of the repeated cross-sectional monitoring survey, *“*Assessing the Impacts of COVID-19 on Mental Health.” Round 1 data collection (14–19 May 2020) occurred at a point when many Canadian provinces began to initiate a “re-opening” phase, following approximately 2 months of restrictions and the initial peak of COVID-19 [26]. Round 2 data collection (14–21 September 2020) coincided with the return of many students to their educational settings, including new online formats for most post-secondary students, as well as an end to the summer months that afforded greater ease of safely gathering in outdoor spaces. Round 3 data collection (22–28 January 2021) followed soon after the winter holiday season, amid rapidly growing case counts and increasingly restrictive public health measures.

For each round, Maru/Matchbox, a national polling agency, distributed online surveys to members of their online Canada Voice Panel, which consists of approximately 125,000 individuals across the country. A variety of measures are used to recruit panelists to reflect hard-to-reach population sub-groups. Potential participants were randomly selected from the panel to receive an invitation to participate in the study. The random sampling utilized Canadian census-informed socioeconomic stratifications (age, gender, household income, province/territory) of panel members with adjustments for response propensity. Data were also statistically weighted according to these stratifications to align with current Canadian Census information. Surveys were anonymous and available in Canada’s two official languages, English and French. At Round 1, 3000 respondents completed the survey (with a 32% invitation-to-response rate); at Round 2, 3027 respondents completed the survey (with a 36% invitation-to-response rate); and at Round 3, 3034 respondents completed the survey (with a 36% invitation-to-response rate). The maximum margin of error for proportions derived from a sample of this size is +/− 1·79% at a 95% level of confidence.

### Ethics

Ethics approval was provided by the Behavioural Research Ethics Board at UBC (H20-01273). All procedures performed were in accordance with ethical standards of this institutional committee and with the 1964 Helsinki declaration and its later amendments. All participants in this study provided online informed consent and received a small honorarium from Maru/Matchbox for completing the survey.

### Measures

Survey items were informed by the Mental Health Foundation’s repeated monitoring survey initiated in March 2020, the development of which was guided by research on the mental health impacts of past pandemics. Original survey items were further shaped by a citizen’s jury participatory process that included people with lived experience of mental health conditions [27]. To adapt the survey to the Canadian context, some items were modified and questions added. For example, following Round 1, our measure for assessing gender was changed to align with current best practices and a question on substance use to cope was added to align with developing data needs. In response to emerging evidence, further refinements were made prior to each subsequent round of surveying, including additional measures. For all rounds, items were included to assess socioeconomic status, gender, sexual orientation, mental health and disability status, and ethnicity. This facilitated analysis of the disproportionate mental health impacts of COVID-19 for a selection of populations known to experience structural vulnerability.

For mental health outcomes, respondents were asked to self-report impacts to their mental health as a result of the COVID-19 pandemic in the past 2 weeks. Change in mental health was assessed by asking “Compared to before the COVID-19 pandemic and related restrictions in Canada, how would you say your mental health is now?” with responses “Slightly worse now” and “Significantly worse now” classified as “Reduced mental health” while responses of “About the same,” “Slightly better now” and “Significantly better now” classified as “Not experiencing reduced mental health.” Emotional responses were assessed by asking “Which of the following emotions have you felt as a result of the COVID-19 pandemic”, with responses selected from a list of common emotions, both positive and challenging. Coping was assessed by asking “Overall, how well do you think you are coping with stress related to the COVID-19 pandemic?” with responses “Not very well” and “Not well at all” classified as “Not coping well” and “Fairly well” and “Very well” classified as “Coping well”. Suicidal ideation and self-harm were assessed by asking “Have you done or experienced any of the following, as a result of the COVID-19 pandemic?” for “Experienced suicidal thoughts/feelings” and “Deliberately hurt myself.” Substance use was assessed by asking respondents to indicate how their use of various substances was impacted by the COVID-19 pandemic with responses of “More” classified as reporting increased use and responses of “No change,” “Less,” “Not applicable” and “Prefer not to say” classified as not reporting an increase in use. The Round 2 and 3 surveys additionally asked respondents if their use of substances increased as a way to *cope* at any point during the pandemic. Full versions of the survey can be found in Supplementary Appendix SA.

### Statistical Analysis

All analyses and reported results were based on data that was statistically weighted according to current Census data for age, gender, region, and income in the adult population of Canada. Descriptive statistics were used to characterize the full sample of respondents at each of the 3 survey rounds. The prevalence (percent with 95% confidence intervals) of individual mental health outcomes were calculated for each sub-group of interest by survey round and presented in clustered bar charts and associated Supplementary Tables using Version 4.0 of the software R. Data from the three survey rounds were then combined into a single dataset. Where a participant had completed more than one round of surveying, their initial survey data were retained, while their subsequent data were removed from the pooled sample. Separate multivariate logistic regression models were then used to quantify the independent risks of experiencing adverse mental health outcomes for each population sub-group adjusting for age, gender, and survey round on the following three empirically informed mental health outcome measures: reduced mental health, experiencing suicidal thoughts, and increased alcohol use. All regression models were conducted using the software SPSS Version 27 and participants who chose not to answer a question were excluded from analyses involving that question via listwise deletion (i.e., regression analyses were based on complete cases) as the amount of missing data was less than 5 percent [28].

## Results

A total of 9,061 survey responses were collected. The Round 1 May 2020 sample (*n* = 3000) was 49.5% female and 29.7% were classified as racialized. The Round 2 October 2020 sample (*n* = 3027) was 48.8% female and 27.6% were classified as racialized. The Round 3 January 2021 sample (*N* = 3034) was 49.8% female and 25.9% were classified as racialized. Additional information on sample demographics can be found in Table 1.

**TABLE 1:** Description of repeated cross-sectional samples for survey rounds 1, 2, and 3 (Assessing the Impacts of COVID-19 on Mental Health, Canada, 2020–2021).

	Round 1 May 2020		Round 2 October 2020		Round 3 January 2021	
	N	%	N	%	N	%
Age group						
18–34 years	840	28.0	838	27.7	839	27.7
35–54 years	1,050	35.0	1,061	35.1	1,057	34.8
55 + years	1,110	37.0	1,128	37.3	1,138	37.5
Gender^a^ ^,^ ^b^						
Cisgender man	1,492	49.7	1,464	48.4	1,479	48.7
Cisgender woman	1,485	49.5	1,478	48.8	1,512	49.8
Non-cisgender	23	0.8	84	2.8	43	1.4
Ethnicity^a^ ^,^ ^c^						
Non-racialized	1938	67.1	1982	69.3	2035	70.6
Racialized	859	29.7	790	27.6	748	25.9
Indigenous	90	3.1	88	3.1	101	3.5
Education completed						
High school or less	421	14.0	475	15.7	426	14.1
Some college or university	498	16.6	563	18.6	451	14.9
College or university graduate	2082	69.4	1989	65.7	2156	71.1
Household income						
Under $25k	253	8.4	196	6.6	239	8.1
$25k-<$50k	497	16.6	549	18.4	497	16.9
$50k-<$100k	990	33.0	983	33.0	971	33.0
$100k +	1,260	42.0	1,252	42.0	1,236	42.0
Pre-existing mental health condition^b^						
Yes	568	19.1	573	19.2	543	18.1
No	2404	80.9	2406	80.8	2463	81.9
Disability^a^						
Yes	299	10.1	342	11.5	303	10.1
No	2672	89.9	2644	88.5	2696	89.9
LGBT2Q + ^a^ ^,^ ^d^						
Yes or unsure	269	9.0	270	9.0	270	8.9
No	2714	91.0	2725	91.0	2749	91.1
Total	3000	100.0	3027	100.0	3034	100.0

a A small number of respondents chose not to answer some questions which reduced the total counts for these variables.

b Gender was assessed by asking participants “Which gender do you most identify with?” For Round 1 respondents, those who responded “Man” were classified as Cisgender men; those who responded “Woman” were classified as Cisgender women; and those who responded “Transgender woman/trans woman”, “Transgender man/trans man”, “Non-binary”, and “Two-Spirit”, were classified as Non-cisgender. This measure was updated in subsequent rounds to better reflect current best practices, and gender was assessed by asking participants which gender they most identify with and “What sex were you assigned at birth?”. Non-binary and transgender identities of Round 2 participants were then determined by comparing current gender identity with sex assignment at birth.

c Racialized status was derived by classifying all respondents as Indigenous if they identified as having Indigenous family origins, even if they identified additional ethnic categories. All respondents who identified a family history of European origins were classified as non-racialized, while respondents who identified as being of one or more of the following origins were categorized as racialized: East Asian, South Asian, Southeast Asian, Latin American, Middle Eastern, African, Other, and Don’t know. Respondents who indicated both a European origin and a non-European origin were classified as being racialized.

d Respondents who said “yes” or “unsure” when asked if they identified as being “lesbian, gay, bisexual, trans, Two-Spirit, queer, etc.” were classified as LGBT2Q+.

### Emotional Responses

**FIGURE 1:**
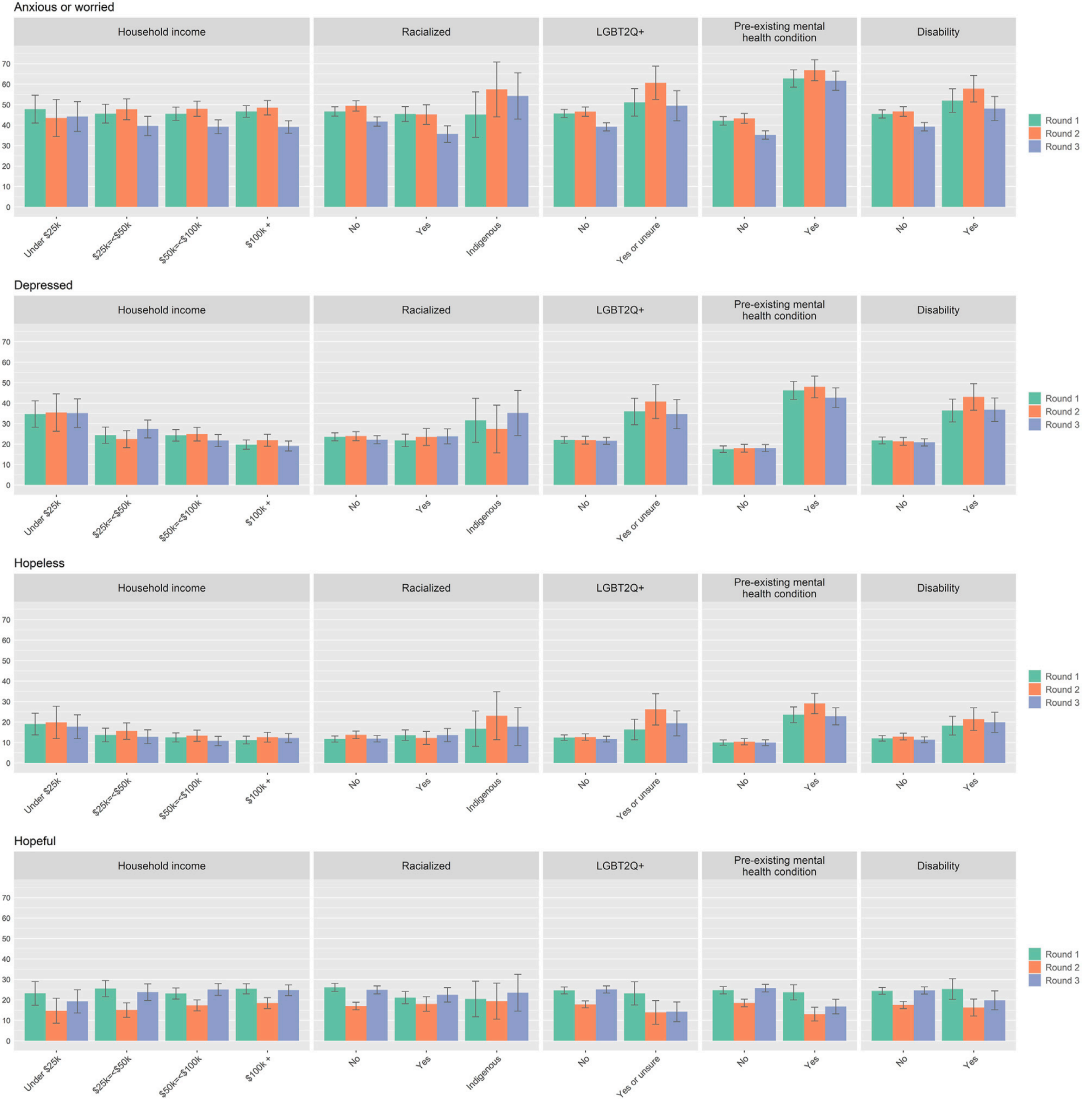
Emotional responses to the COVID 19 pandemic by sub-group for survey Round 1, 2, and 3. Percentages in the bar charts are accompanied by 95% confidence intervals. (Assessing the Impacts of COVID-19 on Mental Health, Canada, 2020–2021).

Considering all three survey rounds together, feeling anxious or worried (44.7%), depressed (23.2%), and, to a lesser extent, hopeless (13.0%), were reported across all population groups. As shown in Figure 1 and in Supplementary Table S1, variation in the extent of emotional responses to the COVID 19 pandemic was observed across groups of respondents, with those who were LGBT2Q+, who had pre-existing mental health conditions, or who had a disability, disproportionately impacted. For example, the proportion of respondents with a pre-existing mental health condition who reported feeling anxious or worried ranged over time from 62.7% in Round 1, to 66.8% in Round 2, and then to 61.7 in Round 3. The proportion without a pre-existing mental health condition who reported these respective emotions was much lower at 42.1% in Round 1, 43.3% in Round 2, and 35.2 in Round 3. A similar pattern was found for feeling hopeful, where 23.7% of those with a pre-existing mental health condition reported feeling hopeful in Round 1, which dropped to 13.1% in Round 2, and increased slightly to 16.7% in Round 3, while a greater proportion of those without a pre-existing mental health condition reported experiencing this positive emotion at 24.7% in Round 1, 18.5% in Round 2, and 25.8% in Round 3.

### Mental Health and Coping

**FIGURE 2:**
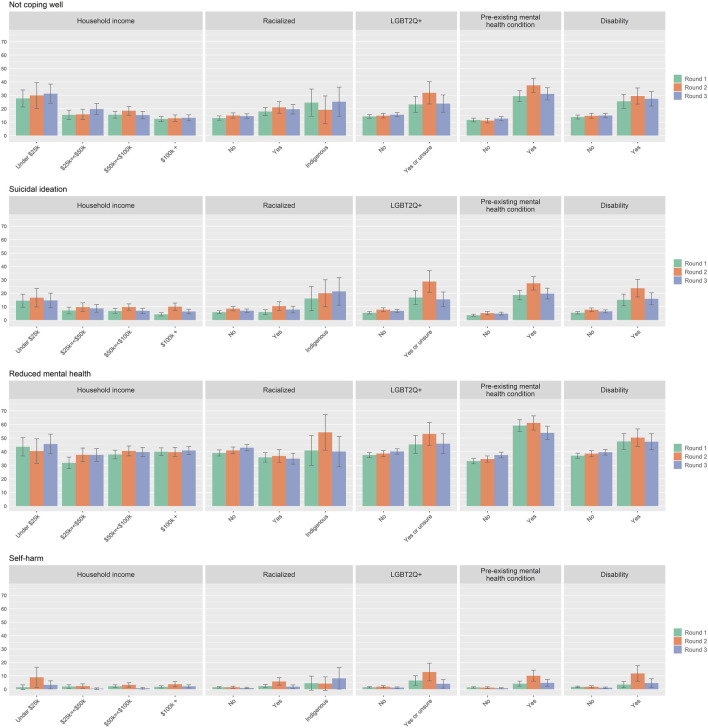
Mental health and coping during the COVID 19 pandemic by sub-group for survey round 1, 2, and 3. Percentages in the bar charts are accompanied by 95% confidence intervals. (Assessing the Impacts of COVID-19 on Mental Health, Canada, 2020–2021).

As shown in Figure 2 and Supplementary Table S2, the prevalence of self-reported reduced mental health and not coping well showed a similar pattern, with higher prevalence of these challenging experiences among those who identify as LGBT2Q+, or who have a pre-existing mental health condition or disability. For example, the prevalence of not coping well among LGBT2Q + respondents ranged between 23.2% in Round 1, increasing to 31.9% in Round 2, and then decreasing to 23.9% in Round 3. The prevalence for non-LGBT2Q + respondents remained lower and relatively stable at 14.3% in Round 1, 14.8% in Round 2, and 15.6% in Round 3. Other groups who showed relatively higher prevalence of not coping well in Round 2 included those with household incomes under $25k (29.9%) and Indigenous respondents (19.2%). The prevalence of suicidal ideation and self-harm showed similar trends to indicators of reduced mental health and not coping well.

### Substance Use

**FIGURE 3:**
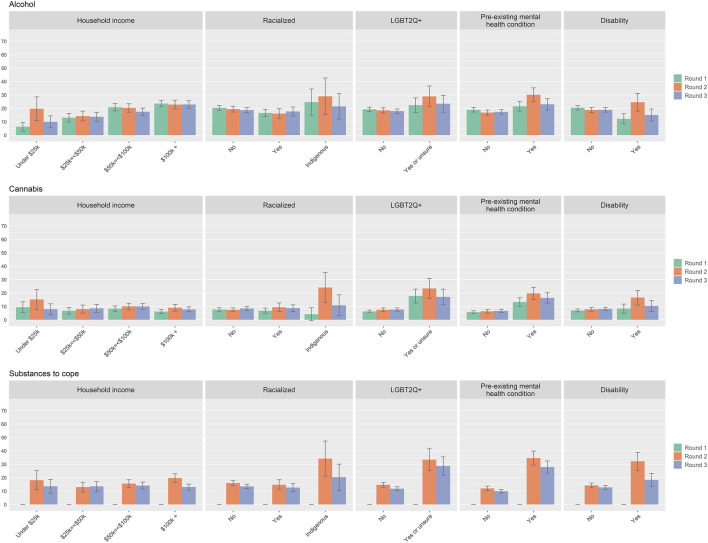
Self-reported increases in alcohol use, cannabis use, and use of substances to cope during the COVID-19 pandemic by sub-group for survey round 1, 2, and 3 (*substances to cope item was not included in Round 1 surveying). Percentages in the bar charts are accompanied by 95% confidence intervals. (Assessing the Impacts of COVID-19 on Mental Health, Canada, 2020–2021).

As with the mental health indicators examined in this study, the prevalence of increased alcohol use, increased cannabis use, and use of substances to cope was also higher among LGBT2Q + respondents, those with a pre-existing mental health condition(s), those with a disability, and Indigenous respondents (See Figure 3; Supplementary Table S3). For example, increased use of cannabis was reported by 13.3% of those with a pre-existing mental health condition in Round 1, 19.8% in Round 2, and 16.3% in Round 3. Those without a pre-existing mental health condition had a lower prevalence of increased cannabis use at 5.8% in Round 1, 6.3% in Round 2, and 6.7% in Round 3.

### Multivariate Logistic Regression Model Results

Of the initial pooled dataset of 9,061 respondents, 2181 (24.1%) had participated in a previous round and were removed, resulting in an independent and non-overlapping sample of 6880 respondents for the regression analyses. Those who participated in multiple rounds were less likely to be aged 55 + years (Chi-square = 229.69, *p* < 0.05), slightly more likely to have a household income greater than $100k (Chi-square = 12.5, *p* < 0.05), slightly more likely to be classified as non-cisgender (Chi-square = 6.20, *p* < 0.05), slightly more likely to be Indigenous or racialized (Chi-square = 12.35, *p* < 0.05) and slightly more likely to be classified as being LGBT2Q+ (Chi-square = 6.77, *p* < 0.05). The results of the multivariate logistic regression models used to quantify the independent risks of experiencing adverse mental health outcomes for each sub-group before (Crude Odds Ratio – OR) and after adjusting (Adjusted OR – AOR) for age, gender, and survey round on the three core mental health measures are presented in Table 2.

**TABLE 2:** Results from logistic regression models quantifying independent effects of sub-group membership on risk of experiencing core mental health outcomes (Assessing the Impacts of COVID-19 on Mental Health, Canada, 2020–2021).

Variables included in multivariate models	Reduced mental health (*n* = 6595)^a^				Suicidal thinking (*n* = 6548)^a^				Increased alcohol use (*n* = 6599)^a^			
	Crude OR	Adjusted OR	95% CI		Crude OR	Adjusted OR	95% CI		Crude OR	Adjusted OR	95% CI	
Age group												
18–34 years	2.01*	1.83*	1.59	2.10	5.27*	3.69*	2.69	5.06	2.50*	2.65*	2.23	3.15
35–54 years	1.46*	1.39*	1.23	1.568	3.65*	3.12*	2.32	4.20	1.81*	1.80*	1.53	2.11
55 + years	Reference				Reference				Reference			
Gender												
Cisgender man	Reference				Reference				Reference			
Cisgender woman	1.68*	1.51*	1.36	1.68	1.17	0.80*	0.64	1.00	1.15*	1.03	0.90	1.17
Non-cisgender	1.28	0.82	0.48	1.38	5.89*	1.81	0.93	3.51	1.33	1.08	0.59	1.97
Survey round												
Round 1	Reference				Reference				Reference			
Round 2	1.14*	1.18*	1.05	1.33	1.44*	1.58*	1.26	1.99	0.93	0.95	0.82	1.10
Round 3	1.09	1.23*	1.07	1.41	0.84	1.07	0.79	1.45	0.86	0.97	0.81	1.15
Ethnicity												
Non-racialized	Reference				Reference				Reference			
Racialized	0.82*	0.77*	0.68	0.87	1.06	0.99	0.77	1.26	0.75*	0.66*	0.56	0.77
Indigenous	0.99	0.71*	0.53	0.97	3.25*	1.84*	1.21	2.78	1.28	1.08	0.77	1.53
Household income												
> $25k	1.04	0.71*	0.58	0.89	2.79*	1.27	0.89	1.82	0.49*	0.39*	0.29	0.54
$25k-$50k	0.80*	0.70*	0.60	0.82	1.41*	1.16	0.86	1.56	0.55*	0.52*	0.43	0.64
$50k-$100k	0.89*	0.78*	0.69	0.89	1.17	0.93	0.72	1.20	0.84*	0.78*	0.67	0.90
$100k plus	Reference				Reference				Reference			
Pre-existing mental health: Yes	2.95*	2.51*	2.18	2.90	6.93*	4.70*	3.73	5.92	1.66*	1.49*	1.26	1.77
LGBT2Q + status: Yes/unsure	1.45*	1.11	0.91	1.35	3.88*	1.76*	1.33	2.34	1.40*	1.16	0.92	1.46
Disability: Yes	1.56*	1.30*	1.09	1.56	3.01*	1.51*	1.13	2.01	0.82	0.88	0.69	1.12

a The variation in sample size for each regression model is due to some respondents choosing not to answer individual questions and being deleted from the models via listwise deletion (i.e., analyses were based on complete cases) as the proportion of missing data was 4.1% for the Reduced mental health model, 4.8% for the Suicidal thinking model, and 4.1% for the Increased alcohol use model.

*Indicates *p* < 0.05.

Compared to those aged 55 years and older, younger age groups were more likely to report a decline in self-rated mental health (e.g., the AOR for those aged 18 to 34 was 1.83, 95% CI 1.59–2.10), more likely to report suicidal thoughts (e.g., the AOR for those aged 18 to 34 was 3.69, 95% CI 2.69–5.06) and more likely to report increased consumption of alcohol (e.g., the AOR for those aged 18 to 34 was 2.65, 95% CI 2.23–3.15). Those who were classified as women were also more likely to report declines in self-rated mental health compared to men (AOR = 1.51, 95% CI 1.36–1.68). However, women were less likely to report suicidal thoughts (AOR = 0.80, 95% CI 0.64–1.00). Among the overall sample, there was also an increase in the odds of reporting suicidal thoughts between Round 1 and Round 2 (AOR = 1.58, 95% CI 1.26–1.99).

With respect to population sub-groups, being racialized (non-Indigenous) was associated with a lower likelihood of reporting a decline in self-rated mental health (AOR = 0.77, 95% CI 0.68–0.87) as well as a lower likelihood of reporting increased alcohol consumption (AOR = 0.66, 95% CI 0.56–0.77). Being Indigenous, however, was associated with a nearly two-fold increase in the odds of experiencing suicidal thoughts (AOR = 1.84, 95% CI 1.21–2.78). Compared to those with household incomes of $100k or greater, having a lower household income was associated with reduced odds of experiencing declines in self-rated mental health (e.g., the AOR for >$25k was 0.71, 95% CI 0.58–0.89) and reduced odds of reporting increase alcohol consumption (e.g., the AOR for <$25k was 0.39, 95% CI 0.29–0.54). Having a pre-existing mental health condition was associated with increased odds of reporting a decline in self-rated mental health (AOR = 2.51, 95% CI 2.18–2.90), increased odds of reporting suicidal thoughts (AOR = 4.70, 95% CI 3.73–5.92), and increased odds of reporting increased alcohol consumption (AOR = 1.49, 95% CI 1.26–1.77). Identifying as LGBT2Q+ was also associated with an increased risk of reporting suicidal thoughts (AOR = 1.76, 95% CI 1.33–2.34). Having a disability was associated with increased risk of endorsing a decline in self-rated mental health (AOR = 1.30, 95% CI 1.09–1.56) and increased risk of experiencing suicidal thoughts (AOR = 1.51, 95% CI 1.13–2.01).

## Discussion

The COVID-19 pandemic has led to significant adverse mental health impacts among the general population, with emerging data indicating that these impacts are distributed along a social gradient wherein populations experiencing structural vulnerabilities are most affected [1, 29, 30]. However, there is a paucity of literature examining the range of pandemic related mental health impacts over time or among population sub-groups who experience disproportionate “risk”. We present data on mental health indicators across population sub-groups from three time points during the pandemic. These data can support evidence-informed responses to address population mental health outcomes and attend to inequities. These findings are consistent with other studies demonstrating persistent and widening disparities in mental health [31, 32], though equity-focused analyses of data at different time points remain limited.

Results from this study illustrate significant inequities in mental health impacts during the COVID-19 pandemic, demonstrating the potential syndemic effects of the pandemic for some structurally vulnerable populations. Consistent with other evidence of differential mental health impacts amid the pandemic [1, 9, 13, 30], our findings illustrate that people who experience structural vulnerability related to pre-existing mental health conditions, disability, LGBTQ2+ identity, or being Indigenous are more likely to report mental health consequences. This includes greater proportions experiencing reduced mental health, challenges in coping, suicidal ideation, self-harm, and feeling hopeless, anxious/worried, and depressed.

These findings were further supported by the results of the logistic regression models indicating significant increases in the risk of experiencing core adverse mental health outcomes, even after adjusting for a wide range of socio-demographic characteristics. Though these findings are aligned with other pandemic-related research on mental health inequities, our data on mental health impacts among racialized populations are, perhaps, counterintuitive. Specifically, like research out of the United States, which has documented comparatively low levels of certain adverse mental health outcomes among Black Americans prior to and during the pandemic (i.e., the “Black-white depression paradox”) [33, 34], our findings show that people who are racialized were less likely to experience adverse mental health outcomes on our core measures compared to non-racialized people during the time period examined. This, despite being inequitably impacted by COVID-19 morbidity and mortality [34, 35]. While the reasons for this outcome are not known, it could be that the cultures and contexts of racialized participants were protective against adverse mental health outcomes during the pandemic. For example, in Canada, racialized populations are more likely than their non-racialized counterparts to live in multigenerational households [36]. This could have contributed to reduced experiences of isolation and loneliness among racialized groups during a time that was characterized by public health restrictions that limited the ability to connect and gather in-person. On the other hand, this finding could also be the product of cultural stigma linked to mental health challenges, which may have led to reporting bias among people from cultural contexts where mental health challenges remain particularly taboo [37, 38]. While our findings provide an overview of the mental health impacts of the pandemic for different groups and populations, they also provide insights into these experiences across the course of the pandemic. For example, there was an independent effect of survey round, whereby Round 2 respondents (September 2020) were more likely to experience a reduction in mental health and increases in suicidal ideation compared to Round 1 respondents (May 2020); however, this was no longer evident in Round 3 (January 2021). Independent analyses, such as those included in this manuscript, are useful from a policy perspective in providing an evidence base for targeted mental health intervention and responsive public policy for ‘high risk’ groups [39] as well as the population more generally. Future research would benefit from more focused investigation on how inequities may change over time within structurally vulnerable groups.

Examinations of previous syndemics—including HIV/AIDS, hepatitis C virus, and tuberculosis—have illustrated that infectious disease outbreaks intersect with underlying structural vulnerabilities and socio-economic and political power structures, leading to inequitable impacts for particular population sub-groups [40, 41]. There are therefore opportunities to mitigate the potential syndemic effects of the COVID-19 pandemic through social and political action to remediate inequities and implement services and supports for structurally vulnerable populations. Indeed, Mendenhall [42] argues that while COVID-19 is experienced as syndemic in many global settings, political and social actions can diminish these effects, as seen in New Zealand as a result of their political response to the crisis. Our findings provide further support for this claim. For example, while many groups who experience structural vulnerability were identified as having increased odds of adverse mental health outcomes, lower household income (<$25k) was associated with reduced risk. In the Canadian context, public policy initiatives such as monthly financial support through the Canada Emergency Response Benefit (CERB) and bans on rent increases and evictions may have lessened the mental health impacts of COVID-19 on those with lower household incomes. Thus, research examining the complexities of the mental health impacts of the pandemic in the context of social and structural contributors is needed. While epidemiological studies often reflect an “individualistic fallacy,” explaining emergent conditions through individual-level covariates [41], research that advances analyses of root systemic contributors to adverse mental health outcomes can more meaningfully support action toward addressing inequities and preventing potential syndemic effects of the COVID-19 pandemic and future health crises.

Responding to disparities in adverse population mental health outcomes amid COVID-19 has been identified as a global priority [43], with data from our analysis further underscoring the need for immediate and meaningful action informed by a population-based approach to mental health. This approach attends to the full spectrum of mental health intervention from promotion through prevention, treatment, and maintenance, and has long been advocated for to respond to the mental health of whole populations [44]. However, Canadian and global mental health efforts thus far have predominantly focused on individually-oriented clinical services, with promotion and prevention receiving less attention and investment [45–48]. The pandemic has highlighted the urgent need for a paradigm shift in the mental health field to respond to the root causes of mental health disparities, including social and economic inequities and discrimination and exclusion of minoritized groups [46]. Building capacity for mental health promotion and prevention, and developing stronger systems for screening and treatment, are needed in response to the considerable mental health impacts of the pandemic [49]. Moreover, the adverse mental health impacts of the COVID-19 pandemic are situated within a broader context of climate, systemic racism, and drug poisoning crises. Referred to by some as “collective traumas,” these crises are having considerable concurrent impacts on mental health among the general population, and structurally vulnerable groups in particular [50, 51]. These intersecting crises and the resultant adverse mental health impacts suggest that, even with the cessation of the pandemic, mental health challenges are unlikely to “resolve” promptly, and thus require ongoing public health and policy responses [32, 52]. Indeed, our investigation spanning three points in the pandemic, illustrates that the mental health impacts are, to date, largely sustained over time. Continued monitoring of these impacts is needed to guide investments to support initiatives aimed at mental health recovery.

### Limitations

This study has noteworthy strengths and limitations. Although the response rates were relatively low (Wave 1: 32%, Wave 2: 36%, and Wave 3: 36%), they are higher than average rates expected for similar online studies of the general population in Canada [53]. Census informed statistical weights were utilized to ensure the sample was representative of the Canadian population by age, gender, region, and income. However, despite efforts to recruit a diverse sample, other demographic characteristics such as ethnicity (including Indigenous identity) were underrepresented, which for some examinations, resulted in large and overlapping confidence intervals. Moreover, as the survey was delivered online, those with technology barriers are not reflected in the sample. Thus, survey respondents may have differed from the overall Canadian population on certain characteristics. However, the repeated cross-sectional study design facilitates continued monitoring of trends over time and enhances confidence in identified outcomes. Additionally, as this study did not use standardized clinical measures of mental health conditions, such as depression or anxiety, we are not able to directly contrast results with other population surveys that utilize these measures. Further, the study did not use validated instruments to assess mental health impacts of the pandemic. However, items were drawn from the Mental Health Foundation survey based on research evidence from past pandemics and input from individuals with lived experience of mental health challenges. These survey items have now been used across 10 + rounds of data collection in the UK and Canada. As results of this study are based on self-report, they are subject to potential recall bias. Furthermore, we lack a pre-pandemic baseline of these measures for comparison. Nonetheless, our findings offer important and timely data to inform actions that are responsive to the mental needs of priority populations and sub-groups.

### Conclusion

While previous studies have largely examined the particular interactions of virus transmission and illness with underlying physical health conditions as salient syndemic effects of COVID-19, our study extends understandings of this syndemic to include the synergistic effects involving the impacts of the COVID-19 pandemic, underlying structural vulnerability, and mental health. This study provides critical evidence that populations who experience structural vulnerability are reporting disproportionately worse mental health due to the COVID-19 pandemic compared to those with relative privilege. Taken together, these findings illustrate the need for an equity-oriented population-based response to mental health that prioritizes prevention and promotion, in addition to treatment and maintenance, to redress disparities and promote mental health and well-being nationally and globally.
