# Supplementary material for: Mental Health Inequities Amid the COVID-19 Pandemic: Findings From Three Rounds of a Cross-Sectional Monitoring Survey of Canadian Adults
Source: Int J Public Health. 2022 Jul 21;67:1604685. doi: 10.3389/ijph.2022.1604685 (PMC9349347; doi:10.3389/ijph.2022.1604685)
Supplement: Supplementary file 1 [file DataSheet2.pdf]

Supplementary Table 1. Emotional responses to the COVID 19 pandemic by sub-group for survey Round 1, 2 and 3.

			Anxious or worried		Depressed		Hopeless		Hopeful	
		Round	%	95% CI	%	95% CI	%	95% CI	%	95% CI
**Household income**	Under $25k	1	47.8	41.0, 54.6	34.7	28.2, 41.1	19.0	13.7, 24.3	23.2	17.4, 29.0
		2	43.5	34.4, 52.5	35.4	26.3, 44.5	19.8	12.0, 27.7	14.7	8.6, 20.8
		3	44.2	36.9, 51.5	35.1	28.1, 42.1	17.7	11.9, 23.6	19.3	13.6, 25.0
	$25k-<$50k	1	45.6	41.0, 50.2	24.3	20.3, 28.3	13.7	10.4, 17.0	25.5	21.6, 29.5
		2	47.7	42.6, 52.8	22.4	18.2, 26.6	15.6	11.6, 19.6	15.0	11.4, 18.6
		3	39.6	34.9, 44.3	27.4	23.0, 31.8	12.8	9.4, 16.2	23.8	19.7, 27.8
	$50k-<$100k	1	45.5	42.3, 48.8	24.3	21.5, 27.1	12.5	10.3, 14.7	23.1	20.4, 25.8
		2	48.0	44.3, 51.7	24.9	21.5, 28.2	13.3	10.6, 16.1	17.3	14.6, 20.0
		3	39.2	35.9, 42.6	21.7	18.8, 24.7	10.8	8.5, 13.0	25.1	22.2, 28.0
	$100k +	1	46.7	43.8, 49.5	19.8	17.5, 22.1	11.2	9.3, 13.1	25.4	23.0, 27.9
		2	48.5	45.0, 52.0	21.9	18.9, 24.8	12.6	10.2, 15.0	18.4	15.8, 21.1
		3	39.1	36.0, 42.1	19.1	16.6, 21.5	12.2	10.0, 14.4	24.8	22.1, 27.4
**Race/ethnicity**	Non-racialized	1	46.7	44.4, 48.9	23.6	21.6, 25.5	11.7	10.2, 13.2	26.1	24.1, 28.1
		2	49.4	46.9, 51.9	23.9	21.7, 26.1	13.8	11.9, 15.6	17.0	15.2, 18.8
		3	41.7	39.4, 44.0	22.2	20.2, 24.1	11.9	10.3, 13.4	24.9	23.0, 26.9
	Racialized	1	45.4	41.8, 49.1	21.8	18.7, 24.9	13.6	11.0, 16.2	21.2	18.2, 24.1
		2	45.2	40.4, 49.9	23.5	19.3, 27.6	12.2	9.1, 15.4	18.0	14.4, 21.6
		3	35.6	31.6, 39.7	23.8	20.1, 27.4	13.6	10.4, 16.8	22.5	19.0, 26.0
	Indigenous	1	45.1	34.0, 56.2	31.6	20.9, 42.4	16.8	8.1, 25.4	20.5	11.8, 29.1
		2	57.4	44.0, 70.8	27.4	15.7, 39.0	23.1	11.3, 34.9	19.4	10.6, 28.2
		3	54.2	42.9, 65.5	35.2	24.2, 46.2	17.8	8.5, 27.0	23.6	14.6, 32.6
**LGBT2Q+**	Yes or unsure	1	51.1	44.4, 57.8	35.9	29.5, 42.4	16.3	11.3, 21.3	23.2	17.5, 28.9
		2	60.6	52.5, 68.8	40.8	32.5, 49.0	26.2	18.6, 33.9	13.9	8.1, 19.7
		3	49.5	42.1, 56.8	34.7	27.6, 41.7	19.4	13.3, 25.5	14.2	9.4, 19.0
	No	1	45.7	43.7, 47.6	22.1	20.4, 23.7	12.4	11.1, 13.7	24.6	23.0, 26.3
		2	46.6	44.3, 48.8	21.9	20, 23.8	12.6	11.1, 14.2	17.8	16.1, 19.5
		3	39.2	37.2, 41.2	21.6	19.9, 23.3	11.6	10.3, 13.0	25.1	23.4, 26.9
**Pre-existing mental health condition**	Yes	1	62.7	58.5, 67.0	46.2	41.8, 50.6	23.6	19.7, 27.4	23.7	20.0, 27.4
		2	66.8	61.6, 71.9	47.9	42.6, 53.2	29.0	24.1, 34.0	13.1	9.7, 16.4
		3	61.7	57.0, 66.3	42.7	37.9, 47.5	22.8	18.7, 27.0	16.7	13.2, 20.3
	No	1	42.1	40.0, 44.1	17.5	15.9, 19.1	10.0	8.7, 11.3	24.7	23.0, 26.5
		2	43.3	40.9, 45.7	18	16.1, 19.9	10.4	8.8, 12.0	18.5	16.7, 20.3
		3	35.2	33.1, 37.2	18.1	16.4, 19.8	9.9	8.5, 11.3	25.8	23.9, 27.6
**Disability**	Yes	1	52.0	46.2, 57.7	36.4	30.9, 42.0	18.2	13.7, 22.8	25.3	20.3, 30.3
		2	57.8	51.3, 64.3	43	36.5, 49.5	21.4	15.9, 26.9	16.3	12.2, 20.4
		3	48.1	42.2, 54.0	36.8	31.1, 42.5	19.9	14.9, 24.8	19.8	15.2, 24.4
	No	1	45.4	43.5, 47.4	21.8	20.1, 23.5	12.1	10.7, 13.4	24.4	22.7, 26.1
		2	46.7	44.3, 49.0	21.3	19.4, 23.3	12.9	11.2, 14.6	17.5	15.8, 19.3
		3	39.2	37.2, 41.3	20.8	19.1, 22.6	11.4	10.0, 12.8	24.6	22.9, 26.4

Supplementary Table 2. Mental health and coping during the COVID 19 pandemic by sub-group for survey Round 1, 2 and 3.

			**Reduced mental health**		**Not coping well**		**Suicidal ideation**		**Self-harm**	
		Round	%	95% CI	%	95% CI	%	95% CI	%	95% CI
**Household income**	Under $25k	1	43.6	36.9, 50.4	27.7	21.3, 34.0	14.5	9.7, 19.3	1.5	-0.2, 3.3
		2	40.5	31.4, 49.5	29.9	20.2, 39.6	16.7	9.8, 23.7	8.9	1.4, 16.4
		3	45.7	38.5, 52.9	31.3	24.1, 38.4	14.7	9.3, 20.2	3.3	0.2, 6.3
	$25k-<$50k	1	31.8	27.5, 36.1	15.4	11.9, 19.0	7.2	4.7, 9.7	2.0	0.7, 3.3
		2	37.7	32.7, 42.7	15.8	12.0, 19.6	9.7	6.5, 12.9	2.4	0.8, 4.0
		3	37.6	32.9, 42.2	19.8	15.7, 24.0	8.8	5.9, 11.6	0.5	-0.1, 1.0
	$50k-<$100k	1	37.9	34.7, 41.1	15.6	13.1, 18.1	6.8	5.1, 8.6	2.3	1.2, 3.4
		2	40.6	36.9, 44.2	18.5	15.2, 21.8	9.7	7.3, 12.2	3.3	1.6, 4.9
		3	39.8	36.4, 43.1	15.3	12.6, 17.9	6.9	5.0, 8.9	0.7	0.1, 1.4
	$100k +	1	40.0	37.2, 42.8	12.4	10.4, 14.4	4.3	3.1, 5.5	1.7	0.9, 2.6
		2	39.7	36.3, 43.2	12.9	10.5, 15.4	10.0	7.3, 12.8	3.8	1.8, 5.7
		3	40.9	37.8, 43.9	13.3	11.0, 15.5	6.4	4.7, 8.2	2.1	1.0, 3.3
**Race/ethnicity**	Non-racialized	1	39.1	36.8, 41.3	13.1	11.5, 14.7	5.9	4.8, 7.0	1.5	0.9, 2.1
		2	41.0	38.5, 43.4	15.0	13.0, 16.9	8.5	6.9, 10.2	1.5	0.7, 2.3
		3	42.9	40.6, 45.2	14.5	12.8, 16.3	7.0	5.7, 8.3	0.9	0.4, 1.5
	Racialized	1	35.8	32.3, 39.3	17.9	14.9, 20.9	6.0	4.1, 7.8	2.3	1.1, 3.6
		2	36.8	32.2, 41.5	21.0	16.7, 25.3	10.4	7.0, 13.7	5.8	3.0, 8.6
		3	34.9	30.9, 38.9	19.6	16.1, 23.2	7.9	5.4, 10.4	1.9	0.6, 3.3
	Indigenous	1	40.9	29.9, 51.9	24.6	14.5, 34.7	16.1	7.1, 25.1	4.6	-0.7, 9.8
		2	54.2	41.1, 67.3	19.2	8.9, 29.6	20.0	9.9, 30.1	4.2	-0.9, 9.3
		3	40.1	28.9, 51.2	25.3	14.4, 36.1	21.4	11.2, 31.7	8.1	0.1, 16.1
**LGBT2Q+**	Yes or unsure	1	45.3	38.7, 52.0	23.2	17.3, 29.2	16.9	11.6, 22.1	6.5	3.0, 10.0
		2	53.1	44.7, 61.4	31.9	23.6, 40.2	28.8	20.7, 36.9	12.9	6.3, 19.4
		3	45.9	38.6, 53.2	23.9	17.4, 30.3	15.5	10.0, 21.0	4.0	0.9, 7.2
	No	1	37.6	35.7, 39.5	14.3	12.9, 15.8	5.5	4.6, 6.4	1.5	1.0, 2.0
		2	38.6	36.4, 40.8	14.8	13.1, 16.6	7.8	6.4, 9.2	1.9	1.1, 2.8
		3	40.1	38.1, 42.1	15.6	14.0, 17.2	6.9	5.8, 8.0	1.2	0.7, 1.8
**Pre-existing mental health condition**	Yes	1	59.2	54.9, 63.5	29.4	25.3, 33.5	18.7	15.1, 22.2	4.2	2.3, 6.0
		2	61.1	56.0, 66.3	37.4	32.2, 42.7	27.4	22.4, 32.4	10.1	6.0, 14.2
		3	53.9	49.0, 58.7	31.1	26.6, 35.7	19.8	15.8, 23.8	4.8	2.3, 7.3
	No	1	33.1	31.2, 35.1	11.7	10.2, 13.1	3.6	2.8, 4.4	1.4	0.9, 2.0
		2	34.6	32.3, 36.9	11.2	9.5, 12.9	5.2	3.9, 6.5	1.2	0.5, 1.9
		3	37.5	35.5, 39.6	12.7	11.1, 14.3	4.9	3.8, 5.9	0.8	0.3, 1.2
**Disability**	Yes	1	47.5	41.8, 53.3	25.6	20.4, 30.7	15.1	10.7, 19.4	3.5	1.2, 5.8
		2	50.4	43.9, 56.8	29.5	23.5, 35.5	23.8	17.2, 30.3	11.8	6.0, 17.6
		3	47.4	41.5, 53.2	27.5	22.0, 32.9	15.9	11.4, 20.4	4.7	1.4, 7.9
	No	1	37.0	35.1, 39.0	13.8	12.4, 15.3	5.5	4.5, 6.4	1.8	1.2, 2.4
		2	38.5	36.2, 40.8	14.8	12.9, 16.6	7.7	6.3, 9.1	1.8	1.0, 2.6
		3	39.6	37.6, 41.7	15.0	13.4, 16.6	6.5	5.4, 7.7	1.1	0.6, 1.7

Supplementary Table 3. Self-reported increases in alcohol use, cannabis use and use of substances to cope during the COVID-19 pandemic by sub-group for survey Round 1, 2 and 3.

			Alcohol		Cannabis		Substances to cope	
		Round	%	95% CI	%	95% CI	%	95% CI
**Household income**	Under $25k	1	6.3	3.1, 9.6	9.4	5.4, 13.4	-	-
		2	19.7	10.8, 28.6	15.2	7.7, 22.6	18.1	11.0, 25.3
		3	10.0	5.5, 14.4	7.9	3.9, 12.0	13.6	8.5, 18.7
	$25k-<$50k	1	13.1	9.9, 16.3	6.7	4.3, 9.2	-	-
		2	14.2	10.6, 17.9	8.0	5.0, 11.0	12.9	9.3, 16.5
		3	13.7	10.4, 17.0	8.5	5.5, 11.5	13.5	9.9, 17.0
	$50k-<$100k	1	20.8	18.1, 23.6	8.3	6.4, 10.3	-	-
		2	20.3	17.1, 23.4	10.0	7.7, 12.4	15.5	12.7, 18.4
		3	17.5	14.8, 20.2	10.0	7.7, 12.3	14.1	11.6, 16.7
	$100k +	1	23.6	21.1, 26.0	6.1	4.7, 7.6	-	-
		2	22.8	19.5, 26.1	9.0	6.5, 11.5	19.7	16.5, 22.9
		3	22.9	20.2, 25.7	7.8	6.0, 9.7	13.0	10.8, 15.2
**Race/ethnicity**	Non-racialized	1	20.3	18.5, 22.2	7.7	6.4, 8.9	-	-
		2	19.4	17.2, 21.6	7.5	6.1, 8.9	16.0	14.0, 18.0
		3	18.7	16.8, 20.5	8.5	7.0, 9.9	13.5	11.8, 15.2
	Racialized	1	16.6	13.8, 19.4	6.8	4.9, 8.8	-	-
		2	16.1	12.4, 19.8	9.4	6.2, 12.6	14.7	11.0, 18.4
		3	17.7	14.3, 21.0	8.7	6.2, 11.2	12.6	9.6, 15.5
	Indigenous	1	24.7	14.8, 34.5	4.2	-0.7, 9.0	-	-
		2	29.0	15.3, 42.6	24.1	13.1, 35.1	34.2	21.1, 47.3
		3	21.5	12.0, 31.0	10.8	3.0, 18.7	20.3	10.5, 30.1
**LGBT2Q+**	Yes or unsure	1	22.4	16.9, 27.8	17.8	12.6, 22.9	-	-
		2	28.9	21.2, 36.7	23.4	15.9, 30.9	33.4	25.2, 41.5
		3	23.4	17.1, 29.7	17.1	11.3, 22.9	28.7	21.8, 35.5
	No	1	19.3	17.7, 20.9	6.3	5.2, 7.3	-	-
		2	18.5	16.5, 20.4	7.5	6.2, 8.8	14.7	12.9, 16.4
		3	17.9	16.3, 19.5	7.7	6.5, 8.9	11.8	10.4, 13.2
**Pre-existing mental health condition**	Yes	1	21.6	17.9, 25.2	13.3	10.2, 16.4	-	-
		2	30.1	24.9, 35.3	19.8	15.2, 24.3	34.6	29.3, 39.8
		3	23.0	18.8, 27.2	16.3	12.5, 20.2	27.9	23.4, 32.4
	No	1	18.9	17.3, 20.6	5.8	4.7, 6.8	-	-
		2	16.8	14.8, 18.8	6.3	5.0, 7.7	12.0	10.3, 13.7
		3	17.4	15.7, 19.1	6.7	5.5, 7.9	9.9	8.5, 11.3
**Disability**	Yes	1	12.2	8.5, 16.0	8.4	5.0, 11.7	-	-
		2	24.6	18.0, 31.1	16.5	11.1, 22	32.2	25.5, 38.9
		3	15.0	10.6, 19.5	10.3	6.3, 14.4	18.3	13.4, 23.2
	No	1	20.4	18.8, 22.0	7.0	5.9, 8.1	-	-
		2	18.7	16.8, 20.7	7.9	6.5, 9.3	14.2	12.4, 16.0
		3	18.9	17.2, 20.6	8.1	6.9, 9.4	12.7	11.2, 14.2
