# Supplementary material for: Mental Health Inequities Amid the COVID-19 Pandemic: Findings From Three Rounds of a Cross-Sectional Monitoring Survey of Canadian Adults
Source: Int J Public Health. 2022 Jul 21;67:1604685. doi: 10.3389/ijph.2022.1604685 (PMC9349347; doi:10.3389/ijph.2022.1604685)
Supplement: Supplementary file 2 [file DataSheet1.PDF]

The 2019 novel coronavirus (2019-nCoV), otherwise known as COVID-19, is an infectious disease that has resulted in a global pandemic. Throughout this questionnaire, we will refer to the disease as COVID-19.

For the following questions, please think about yourself, members of your household, or other family members who have been affected by the COVID-19 pandemic.

A "Prefer not to answer" option has been provided if you do not wish to share your experiences on a particular question.

What's your age?

In which province or territory do you currently live?

Alberta
British Columbia
Manitoba
New Brunswick
Newfoundland and Labrador
Northwest Territories
Nova Scotia
Nunavut
Ontario
Prince Edward Island
Quebec
Saskatchewan
Yukon

Do you live in an urban or rural environment?

Urban
Rural

Please select the highest level of education you have completed.

Elementary/grade school
Some high school
High school graduate
Some college / technical school
Completed college / technical school
Some university
University undergraduate degree
Some post-graduate school
Post-graduate degree

What is your marital status?

Single, never married
Common law
Civil partnership
Married
Separated
Divorced

Widowed
Engaged

Please indicate the category which best describes your total household annual income before taxes.

Less than $25,000
$25,000 to less than $35,000
$35,000 to less than $50,000
$50,000 to less than $75,000
$75,000 to less than $100,000
$100,000 to less than $125,000
$125,000 to less than $150,000
$150,000 to less than $250,000
$250,000 to less than $500,000
$500,000 or more
Don't know/prefer not to say

SECTION 2: COVID QUESTIONS

The COVID-19 pandemic has had substantial impacts on employment. Which of the following best describes your current employment status? (Please select all that apply)

Working full time (30 or more hours per week)
Working part time (fewer than 30 hours per week)
Full time student (e.g. school, college, university, job training)
Part time student (e.g. school, college, university, job training)
Not working (e.g. parental leave, disability, medical leave, etc.)
Not working due to personal preference because of COVID-19
Volunteer (unpaid)
Retired
Unemployed (due to COVID-19)
Unemployed (prior to COVID-19)
Underemployed (loss of hours due to COVID)
Other
Prefer not to answer [EXCLUSIVE]

The job that I am currently working in has been deemed as an essential service during the COVID-19 pandemic (i.e., your position is identified by your provincial government as an essential service and you have continued to work in your role throughout the pandemic).

Yes
No
Prefer not to answer

Please select the category that **BEST** describes your professional role:

Health and health services
Law enforcement, public safety, first responder
Vulnerable population service provider (e.g., community outreach, substance use and addiction services)
Education (K-12)
Education (post-secondary)
Education (early childhood)
Food and agriculture service provider (farming, food processing, grocery, hardware)
Transportation
Industry and manufacturing
Communications and information technology
Financial institutions
Retail
Other [ANCHOR]
Prefer not to answer [ANCHOR]

In general, would you say your mental health is:

Excellent
Very good
Good
Fair
Poor

Compared to before the COVID-19 pandemic and related restrictions in Canada, how would you say your mental health is **now**?

Significantly better now
Slightly better now
About the same
Slightly worse now
Significantly worse now
Prefer not to answer

Which of the following applies to how you have been affected by COVID-19 at any point during the pandemic? (Please select all that apply)

I have been tested for COVID-19 and had a positive result
I have been tested for COVID-19 and had a negative result
Someone else in my household has tested positive for COVID-19
Someone else in my household has tested negative for COVID-19
A family member/loved one living at a different address has tested positive for COVID-19
I have self-isolated with symptoms of COVID-19
My household has self-isolated because someone else in my household had symptoms of COVID-19
My household has self-isolated due to contact with someone else who had symptoms of COVID-19

My household has self-isolated due to recent travel
A family member/loved one living at a different address has self-isolated with symptoms of COVID-19
As part of my work I have worked directly with individuals who have tested positive for COVID-19
I have been hospitalized due to COVID-19
Someone in my household has been hospitalized due to COVID-19
A family member/loved one living at a different address has been hospitalized due to COVID-19
A family member/loved one is living at a long-term care facility that had cases of COVID-19
Someone in my household has died due to COVID-19
A family member/loved one living at a different address has died due to COVID-19
None of these [ANCHOR] [EXCLUSIVE]
Don’t know [ANCHOR] [EXCLUSIVE]
Prefer not to answer [ANCHOR] [EXCLUSIVE]

Which of the following emotions have you felt as a result of the COVID-19 pandemic in the **past 2 weeks**? (Please select all that apply)

Afraid
Panicked
Anxious or worried
Empathetic
Indifferent
Hopeful
Hopeless
Ashamed
Guilty
Lonely or isolated
Unprepared
Sad
Angry
Stressed
Bored
Inspired
Depressed
Calm
Comfortable
Content
Secure
None of these [ANCHOR] [EXCLUSIVE]
Don’t know [ANCHOR] [EXCLUSIVE]
Prefer not to answer [ANCHOR] [EXCLUSIVE]

Have you been stressed or worried about any of the following as a result of the COVID-19 pandemic in **the past 2 weeks?** (Please select one option on each row)

Yes

No
Don’t know /Not applicable/ Prefer not to say

Financial concerns (e.g. going into debt, ability to pay bills, long-term economic impacts, etc.)
Being unable to access government benefit payments, not being eligible for benefits, or losing benefits
Losing my job / loss of my job
Being able to cope with uncertainty (e.g. not knowing what will happen)
Becoming ill with the virus
Having no-one to care for me as a result of becoming ill with the virus
Not being able to care for friends and family as a result of becoming ill with the virus
Not being able to care for friends and family due to physical distancing
Passing COVID-19 on to someone else if I became infected
The spread of new variants/mutations of the virus (e.g., UK COVID-19 variant)
Experiencing discrimination if I contract COVID-19
Being vulnerable because other people are not following public health orders
Being vulnerable because of an existing medical condition, age, etc.
Being separated from friends and family
Being able to cope with physical/social distancing (including concerns when needing to leave my residence for groceries, exercise, health care, etc.)
Having enough food to meet my household’s basic needs
My education or career training being interrupted
Looking after my children while continuing to work
Making my existing mental health problems worse
Worrying about how the mental health of my child(ren) will be affected by the pandemic
My child contracting COVID-19 at childcare/daycare
My child contracting COVID-19 at school
My child bringing the virus into the household and infecting other people
Physical distancing measures negatively impacting my child’s ability to learn
Being able to provide educational support while homeschooling my child(ren)
Ability of my child to learn at home while homeschooling
Physical distancing measures negatively impacting my child’s ability to play and connect with friends
Contracting the virus in my work setting
Being able to access a vaccine
The safety or effectiveness of a COVID-19 vaccine
The duration of protection provided by the vaccine(s)
Worrying about the impacts of the ongoing “second wave” of COVID-19
Worrying about the long term mental health impacts resulting from the stress of the pandemic
Experiencing relationship challenges with my partner
Being safe from physical or emotional domestic violence
Fear of getting severely sick or dying
Fear of a family member/loved one getting severely sick or dying

During the past **2 weeks**, how would you rate your sleep quality overall?

Very good

Fairly good
Fairly bad
Very bad

You and other household members worried that food would run out before you got money to buy more. Was that often true, sometimes true, or never true in the last 12 months?

Often true
Sometimes true
Never true
Don't know/prefer not to answer

Since the onset of the COVID-19 pandemic and related restrictions in Canada, have you or any members of your household accessed food-based community programs to get food? (please select all that apply)

Food Bank
Soup Kitchens/Free Meal programs
Meal or food programs from a school
Community Kitchen program
Community Garden
Food voucher program (e.g., receiving gift cards for food from a charitable organization)
Food delivered by a community program
Asking friends or family for help with food
Other [ANCHOR]
No – I haven't accessed any food programs [EXCLUSIVE] [ANCHOR]

Overall, how well do you think you are coping with stress related to COVID-19 pandemic?

Very well
Fairly well
Not very well
Not well at all
Don't know
Prefer not to say
Not applicable – I have not experienced any stress related to COVID-19

Which of the following have helped you to cope with **stress** related to the COVID-19 pandemic in the **past 2 weeks**? (Please select all that apply)

Connecting with those in my household
Connecting with my family or friends **virtually** (e.g., phone, video chat, etc.)
Connecting in-person with friends or family in my "bubble"
Enjoying outdoor activities with friends or family
Connecting with a mental health worker or counsellor **virtually** (e.g. via phone, video chat, etc.)
Having a supportive employer
Spending time with my pet(s)

Receiving in-person mental health supports
Accessing virtual mental health resources (e.g. online cognitive behavioural therapy, etc.)
Maintaining a healthy lifestyle (e.g. balanced diet, enough sleep, exercise, etc.)
Keeping up to date with relevant information (e.g. TV news, newspapers, online information, etc.)
Limiting my exposure to the news about COVID-19
Limiting exposure to social media (e.g. Facebook, Instagram, Snapchat, Twitter etc.)
Increasing my use of social media (e.g. Facebook, Instagram, Snapchat, Twitter etc.)
Contacting a support group (i.e., where members with the same issues can come together for sharing coping strategies, to feel more empowered and/or for a sense of community)
Going for a walk/exercise outside
Exercising in my home
Doing a hobby
Learning or doing something new
Volunteering to help
Accessing federal government benefits and supports (e.g., Canada Emergency Response Benefit, Canada Emergency Wage Subsidy, Canada Emergency Student Benefit, Employment Insurance, etc.)
Accessing provincial government supports (e.g., emergency benefits for workers)
Going to local businesses that are open (e.g., restaurants, hair salons/barber, clothing stores)Other [open] please specify [anchor]
Don’t know [ANCHOR] [EXCLUSIVE]
Nothing has helped me to cope with my stress related to COVID-19 [ANCHOR] [EXCLUSIVE]
Not applicable – I don’t feel stressed [ANCHOR] [EXCLUSIVE]

If you have experienced mental health challenges at any point during the pandemic, have you used virtual (online or phone-based) mental health services and supports (e.g., counselling, online cognitive behavioural therapy, mental health coaching sessions)?

Yes
No
Not applicable. I haven’t experienced a mental health challenge during the pandemic
Prefer not to say

Virtual mental health supports are receiving substantial investment to help people cope with mental health challenges during the pandemic. In order to inform better programs, could you please indicate why you did not access virtual mental health supports. (Select all that apply)

Stigma
Didn’t feel I needed help
Privacy concerns
I don’t think they would be helpful
I didn’t know these supports were available
Necessary equipment not available (e.g., computer, smart phone)
Connectivity issues (e.g., no/slow internet connection)
Competing demands on my time
I prefer in-person health care supports
Other (Please specify) [ANCHOR]

Are you aware of any of the following virtual mental health supports in Canada?

Yes
No
Don’t know /Not applicable/ Prefer not to say

BounceBack
Wellness Together Canada
Strongest Families
WellCan
MindBeacon
Other [ANCHOR]

Please indicate how your use of any of the following has been impacted by the COVID-19 pandemic? (Please select one option on each row)

More
Less
No change
Not applicable
Prefer not to say

Drinking alcohol
Use of tobacco products (e.g. cigarettes, cigars, chewing tobacco, vaping, etc.)
Use of cannabis products
Use of prescribed medication
Use of other psychoactive substances (e.g., cocaine, heroin)
Gambling
Eating/Food consumption
Screen time
Compulsive online shopping (e.g. buying things you don’t really need)

Has your use of substances increased as a way to cope at any point during the pandemic?

Yes
No
Prefer not to say

SECTION 3: Self-harm section

The following questions are on the topic of self-harm and suicidal thoughts. We understand this can be a sensitive topic, so please remember that your answers are anonymous. If you are in crisis, please call 1-833-456-4566 toll free (In QC: 1-866-277-3553), 24/7 or visit www.crisisservicescanada.ca

Have you done or experienced any of the following, as a result of the COVID-19 pandemic in the **past 2 weeks**? (Please select one option on each row)

Yes
No
Prefer not to say

Experienced suicidal thoughts/feelings
Deliberately hurt myself
Worried about someone close to me experiencing suicidal thoughts/feelings or deliberately hurting themselves

How often have you experienced or done each of the following as a result of the COVID-19 pandemic in the **past 2 weeks**? (Please select one option on each row)

Once a day or more often
Nearly everyday day
A few times a week
Passing thoughts
Don’t know
Prefer not to say

Experienced suicidal thoughts/feelings
Deliberately hurt myself
Worried about someone close to me experiencing suicidal thoughts/feelings or deliberately hurting themselves

SECTION 4: DEMOGRAPHICS

What sex where you assigned at birth?

Male
Female

We know that gender has important consequences for health and how we are treated by different individuals and institutions. Which gender do you most identify with?

Female
Male
Non-binary
Two-Spirit
Not listed
Prefer not to answer

What is your ethnic origin? Ethnic origin refers to the ethnic or cultural origins of your ancestors (who are usually more distant than a grandparent) (Check all that apply)

Indigenous origins (for example, First Nations, Inuit, Métis)
East Asian origins (for example, Chinese, Japanese, Korean)
South Asian origins (for example, Indian, Punjabi, Pakistani)
Southeast Asian origins (for example, Filipino, Thai, Vietnamese)
Latin American origins (for example, Brazilian, Cuban, Bolivian)
European origins (for example, British, German, Russian)
Middle Eastern origins (for example, Iranian, Iraqi, Afghan)
African origins (for example, Nigerian, Ghanaian, Zimbabwean)
Other (please specify) ______________________________ [ANCHOR]
Don’t know [EXCLUSIVE] [ANCHOR]
Prefer not to answer [EXCLUSIVE] [ANCHOR]

We know that people of different races do not have significantly different genetics. But our race still has important consequences, including how we are treated by different individuals and institutions. Which race category best describes you? Check all that apply:

Black (African, Afro-Caribbean, African Canadian descent)
East Asian (Chinese, Korean, Japanese, Taiwanese descent)
Southeast Asian (Vietnamese, Cambodian, Thai, Filipino, Indonesian, other Southeast Asian descent)
Indigenous (First Nations, Métis, Inuk/Inuit descent)
Latino (Latin American, Hispanic descent)
Middle Eastern (Arab, Persian, West Asian descent (e.g. Afghan, Egyptian, Iranian, Lebanese, Turkish, Kurdish)
South Asian (East Indian, Pakistani, Bangladeshi, Sri Lankan, Indo-Carribean descent)
White (European descent)
Another race category (Includes values not described above)
Do not know
Prefer not to answer

Do you identify as being LGBT2Q+ (lesbian, gay, bisexual, trans, two-spirit, queer, etc.)?

Yes
No
Unsure
Prefer not to answer

Do you identify as a person with a disability?

Yes
No
Prefer not to answer

Do you identify as a person who has a pre-existing (prior to COVID-19) mental health condition?

Yes
No
Prefer not to answer

The COVID-19 pandemic is impacting people with different mental health conditions in various ways. To help us gain a better understanding of this, please identify the pre-existing mental health condition that you experience (check all that apply).
Mood disorder (e.g., depression, bipolar disorder)
Anxiety disorder
Personality disorder
Psychotic disorder (e.g., schizophrenia)
Eating disorder
Trauma-related disorder (e.g., post-traumatic stress disorder)
Substance use disorder
Other
Prefer not to answer

Which of the following best describes your Canadian citizenship status?

Canadian citizen by birth
Canadian citizen by naturalization
Landed immigrant/Permanent resident
Refugee
Not a citizen
Prefer not to say

How many children (under 18 years of age) reside in your household?

0
1
2
3+

Which of the following best describes your parental/guardian status?

Not a parent / guardian
Parent / guardian (to a child of any age)
Prefer not to say

What age group is/are your child/children? (Please select all that apply)

4 years and under
5-11 years
12-17 years
18 years and over

Are you a single parent?

Yes
No
Prefer not to say

Which of the following best describes the childcare/daycare or school situation of your child/children?

One (or more) of my child/ren is attending childcare/daycare
One (or more) of my child/ren is attending school
One (or more) of my children is homeschooled due to the COVID-19 pandemic
One (or more) of children is homeschooled independent of the COVID-19 pandemic
Other
Prefer not to say

Compared to before the COVID-19 pandemic and related restrictions in Canada, how would you say the mental health **of your child/children is now**?

Significantly better now
Slightly better now
About the same
Slightly worse now
Significantly worse now
It is affecting my children differently (some feel better/some feel worse)
Prefer not to answer

Which do you think has helped your child(ren) cope with **stress** related to COVID-19 pandemic in the **past 2 weeks**? (Please select all that apply)

Connecting with family who live outside our home **virtually** (e.g. phone, video chat, text etc.)
Connecting with friends **virtually** (e.g. phone, video chat, text etc.)
Contacting a **school or community-based** mental health worker or counsellor **virtually** (e.g. via phone, video chat, etc.)
Receiving **in-person** mental health supports
Accessing virtual mental health resources through medical professionals (e.g. online cognitive behavioural therapy, etc.)
Accessing virtual educational or self-help mental health resources through websites, apps, or phone (e.g., Headspace, KidsHelpPhone)
Participating in a virtual child/youth support group
Maintaining a healthy lifestyle (e.g. balanced diet, enough sleep, exercise, etc.)
Maintaining family routines (e.g., family meals, bedtime routines)
Keeping up to date with relevant information (e.g. TV news, newspapers, online information, etc.)
Limiting their exposure to the news about COVID-19
Limiting their exposure to social media (e.g. Facebook, Instagram, Snapchat, Twitter etc.)
More time for social media use (e.g. Facebook, Instagram, Snapchat, Twitter etc.)

Going for a walk/exercise outside
Exercising in our home
Spending time with pet(s)
Playing outdoors
Playing inside (e.g., games, toys, telling stories)
Doing a hobby (e.g., music, reading, arts & crafts)
Volunteering to help
Connecting **in person** with friends
Connecting **in person** with teachers/childcare providers
Connecting **in person** with friends outside of school/childcare
Other (please specify)
Don’t know
Not applicable
Nothing has helped my child(ren) to cope with stress related to COVID-19

Please indicate how your interactions with your child(ren) have been impacted by the COVID-19 pandemic. (Please select one option on each row)

More
Less
No change
Not applicable
Prefer not to say

Having quality time with my child(ren)
Feeling closeness with my child(ren)
Showing love or affection to my child(ren)
Observing resilience (strength and perseverance) in my child(ren)
Disciplining my child(ren)
Conflicts with my child(ren)
Using harsh words with my child(ren)
Yelling/shouting at my child(ren)
Spanking or hitting my child(ren)

How many people reside in your household?

[RANGE: 1 TO 99]

Which of the following best describes your living arrangements? (Please select all that apply)

I live alone
Living with a spouse or partner
Living with friend(s) or housemate(s)
Living with siblings
Living with my child(ren) who are over 18
Living with my child(ren) who are under 18

Living with other adult family members (e.g., parents, grandparents)
Living with grandchildren
Other
Prefer not to answer
None of the above
